# A second-order dynamical system for solving inverse quasi-variational inequalities and its application

**DOI:** 10.1371/journal.pone.0344815

**Published:** 2026-03-19

**Authors:** Ting Gan, Vajahat Karim Khan, Md. Kalimuddin Ahmad, Qing-Bo Cai

**Affiliations:** 1 School of Foreign Languages, Jimei University, Xiamen, China; 2 Department of Mathematics, Aligarh Muslim University, Aligarh, Uttar Pradesh, India; 3 School of Mathematics and Computer Science, Quanzhou Normal University, Quanzhou, P. R., China; Khalifa University of Science and Technology, UNITED ARAB EMIRATES

## Abstract

In this paper, we focus on a second-order dynamical system designed to solve inverse quasi-variational inequalities (IQVIs) in Hilbert spaces, focusing on strongly monotone operators under Lipschitz continuity assumptions. This study establishes the existence and uniqueness of strong global solutions under standard conditions, ensuring the robustness of the proposed system. Furthermore, we derive a discrete-time formulation of the dynamical system, which leads to a relaxed inertial projection algorithm that achieves linear convergence under suitable parameter conditions. Beyond theoretical analysis, stability is verified using a Lyapunov function. Finally, numerical experiments confirm the theoretical results and provide deeper insight into inverse quasi-variational inequality problems within the framework of dynamical systems.

## 1 Introduction

The real *n*-dimensional Euclidean space with an inner product ⟨·,·⟩ and its corresponding norm ‖·‖ is denoted by ${\mathbb{R}}^{n}$. Assume that $\phi :{\mathbb{R}}^{n}\to {\mathbb{R}}^{n}$ is a continuous operator and $𝔇\subset {\mathbb{R}}^{n}$ be a nonempty, closed, and convex set. We consider the standard formulation of the variational inequality problem (VIP), which requires determining $u^{*}\in 𝔇$ satisfying the following condition.


$$\langle \phi (u^{*}),u-u^{*}\rangle \geq 0,\;\forall u\in 𝔇.$$ (1)

Variational inequality problems (VIPs) have been widely studied in both theoretical and applied fields, including optimization, complementarity problems, saddle-point (min-max) problems, Nash equilibrium models, and fixed-point theory [1]. Consequently, various solution approaches, particularly projection-type methods, have been extensively explored (see, for example, [2–7]). From [1], we use the well-established projection theorem, where we conclude that the solution of VI(ϕ,𝔇) is ${𝔲}^{*}\in 𝔇$ defined in (1) ⇔ it has the following projection equation, for a fixed constant μ>0:


$$u^{*}=P_{𝔇}[u^{*}-\mu \phi (u^{*})].$$ (2)

A key extension of the variational inequality problem is the inverse formulation of VI(ϕ,𝔇). Suppose that $\Psi :{\mathbb{R}}^{n}\to {\mathbb{R}}^{n}$ is a single-valued mapping. If the inverse function $𝔴=\Psi^{-1}(u)=\phi (u)$ exists, then VIP can be formulated as an inverse variational inequality problem (IVIP), denoted as IVIP(Ψ,𝔇), which requires determining ${𝔴}^{*}\in {\mathbb{R}}^{n}$ satisfying the following conditions


$$\Psi ({𝔴}^{*})\in 𝔇,\;\text{and}\;\langle \nu -\Psi ({𝔴}^{*}),{𝔴}^{*}\rangle \geq 0,\;\forall \nu \in 𝔇.$$ (3)

While inverse variational inequality problems (IVIPs) appear in various fields, including economic equilibrium models [8] and traffic network analysis [9], there are relatively few theoretical and numerical approaches available for solving the IVI(Ψ,𝔇) (3). It is equivalent to finding a solution to the following projection equation,


$$\Psi ({𝔴}^{*})=P_{𝔇}[\Psi ({𝔴}^{*})-\mu {𝔴}^{*}],$$ (4)

where *μ* is any fixed constant and $P_{𝔇}:{\mathbb{R}}^{n}\to 𝔇$ is the nearest point projection from ${\mathbb{R}}^{n}$ onto 𝔇 defined by


$$P_{𝔇}(𝔴)=\arg\min_{v\in 𝔇}\|𝔴-v\|,\;𝔴\in {\mathbb{R}}^{n}.$$ (5)

In 2019, Zou et al. [10] introduced a dynamical system, referred to as a neural network, to solve IVI(Ψ,𝔇) (3) by formulating the following system:


$$\dot{𝔴}=\sigma \left[P_{𝔇}(\Psi (𝔴)-\mu 𝔴)-\Psi (𝔴)\right],$$ (6)

where $\dot{𝔴}=\frac{d𝔴}{dt}$, $P_{𝔇}$ is defined in (5) and σ>0 be a fixed parameter. He et al. [11] formulated the IVIP(Ψ,𝔇) and developed a projection-based iterative algorithm for its solution, applying their approach to solve many equilibrium problems. IVIP(Ψ,𝔇) have significant applications in engineering science, including transportation and telecommunication networks [12].

Several researchers have devoted their attention to the generalization of IVIPs, leading to the important class of problems known as inverse quasi-variational inequality problems [13,14]. This work addresses the inverse quasi-variational inequality problems (IQVIPs), where the objective is to determine a point $w^{*}\in {\mathbb{R}}^{n}$ such that


$$\Psi (w^{*})\in \Phi (w^{*})\;\text{and}\;\langle w^{*},v-\Psi (w^{*})\rangle \geq 0\;\forall v\in \Phi (w^{*}),$$ (7)

where $\Psi :{\mathbb{R}}^{n}\to {\mathbb{R}}^{n}$ be a single-value mapping and $\Phi :{\mathbb{R}}^{n}\to 2^{{\mathbb{R}}^{n}}$ be a set-valued mapping.

It is evident that when Φ(𝔴)=𝔇 for all $𝔴\in {\mathbb{R}}^{n}$, the inverse quasi-variational inequality problem (IQVIP) described above simplifies to the IVIP. Numerous analytical and computational methods have been developed by various researchers to address this IQVIP. Dynamical systems have become a focal point of study in recent years for addressing a range of optimization problems, including variational inequalities, monotone inclusions, and fixed point problems (see, e.g., [15–17]). In addition, numerous algorithms have been proposed to solve associated VIPs and monotone inclusions through discrete forms of dynamical systems.

Han et al. [8] investigated IQVIP formulated in (7), establishing the existence of a solution through the application of a fixed-point theorem and the Fan–Knaster–Kuratowski–Mazurkiewicz (KKM) Lemma. They also demonstrated its relevance to road pricing models, where the goal of traffic authorities is to regulate environmental impact within acceptable limits. This existence result was later generalized by Dey et al. [18].

Aussel et al. [13] proposed a new framework for IQVIP in 2013, establishing both local and global error bounds through the use of a gap function to highlight its practical utility. To illustrate the effectiveness of the model, they examined a road pricing scenario where environmental impacts from traffic flow are incorporated into the determination of road taxes. In subsequent years, this line of research has been further developed and generalized by numerous authors, including the extension to vector-valued IQVIPs (see [19,20] and the references therein).

In 2024, Dey S. and Reich S. [14] took the dynamical system to solve for inverse quasi-variational inequalities (7) by formulating the following system:


$$\dot{𝔴}=\sigma \left[P_{\Phi (𝔴)}(\Psi (𝔴)-\mu 𝔴)-\Psi (𝔴)\right],$$ (8)

where $\dot{𝔴}=\frac{d𝔴}{ds}$, $P_{\Phi (𝔴)}$ is defined in (5) and σ>0 be a parameter, which is fixed. Several first- and second-order dynamical systems have been introduced to solve different types of variational inequalities, utilizing their stability properties and convergence guarantees [16,21,22]. Motivated by the above findings, we propose a second-order dynamical system to solve IQVIPs (7). The state variable 𝔴(s) evolves according to the following second-order dynamical system with initial conditions:


$$\left\{ \begin{array}{c} \ddot{𝔴}(s)+\tau (s)\dot{𝔴}(s)+\sigma (s)[\Psi (𝔴)-P_{\Phi (𝔴)}(\Psi (𝔴)-\mu 𝔴)]=0,\;s\geq 0,\\ 𝔴(0)={𝔴}_{0},\;\dot{𝔴}(0)=v_{0}. \end{array}\right.$$ (9)

where $\ddot{𝔴}=\frac{d\dot{w}}{ds}$, $\tau :{\mathbb{R}}^{+}\cup \{0\}\to {\mathbb{R}}^{+}\cup \{0\}$ and $\sigma :{\mathbb{R}}^{+}\cup \{0\}\to {\mathbb{R}}^{+}\cup \{0\}$ are Lebesgue measurable functions. If we assume that the set-valued mapping $\Phi :{\mathbb{R}}^{n}\to 2^{{\mathbb{R}}^{n}}$ associated with the IQVIP (7) has nonempty, closed, and convex values. Then a point ${𝔴}^{*}$ solves the IQVIP (7) if and only if it satisfies the projection equation


$$\Psi ({𝔴}^{*})=P_{\Phi ({𝔴}^{*})}(\Psi ({𝔴}^{*})-\mu {𝔴}^{*}).$$ (10)

The existence and uniqueness of the solution to the IQVIP follow from the structural properties of the operator Ψ and the projection mapping associated with Φ; see [14] for related results. Moreover, ${𝔴}^{*}$ is a solution of the IQVIP (7) if and only if it is an equilibrium point of the proposed dynamical system (9), that is, the constant function $𝔴(s)\equiv {𝔴}^{*}$ is a trajectory of the system. Assuming that Ψ is Lipschitz continuous ensures the global existence and uniqueness of system trajectories, while strong monotonicity of Ψ guarantees that all trajectories of the system (9) converge globally to the unique solution of the IQVIP (7). In these applications, real-time solutions are often required; however, conventional iterative methods can be computationally expensive due to the problem’s complexity and high dimensionality.

Dynamical systems have recently attracted considerable attention for solving optimization problems, variational inequalities, monotone inclusions, and fixed-point problems [22–30]. In particular, neurodynamic and inertial-type approaches have been widely developed for classical variational inequality problems. However, their extension to inverse quasi-variational inequalities (IQVIs) remains limited. Most existing methods focus on first-order dynamics or static projection schemes and do not provide a second-order dynamical framework that simultaneously guarantees the existence and uniqueness of global solutions, Lyapunov-based stability of the continuous-time system, and linear convergence of the associated discrete algorithm.

Motivated by these limitations, this paper proposes a second-order dynamical system to solve IQVIPs (7). The proposed framework addresses these challenges by providing a robust and well-posed model under strong monotonicity and Lipschitz continuity assumptions. The use of a second-order formulation introduces a momentum term that accelerates convergence toward the equilibrium point, leading to faster, smoother, and more stable trajectories compared to first-order systems. Consequently, stronger convergence guarantees, such as linear or fixed-time convergence, can be established.

The main contributions of this paper are summarized as follows:

- We propose a second-order dynamical system for solving inverse quasi-variational inequality problems (7) in Hilbert spaces.
- We establish the existence and uniqueness of strong global solutions under strong monotonicity and Lipschitz continuity assumptions.
- A Lyapunov function is constructed to prove the global asymptotic stability of the proposed dynamical system (9).
- A relaxed inertial projection algorithm is derived from the continuous system (9) and shown to converge linearly to IQVIPs (7).
- Numerical experiments validate the theoretical results and demonstrate the effectiveness of the proposed method.

## 2 Definitions and preliminaries

We include in this section some required definitions and lemmas.

**Definition 1.** [31] *The operator* $\Psi :{\mathbb{R}}^{n}\to {\mathbb{R}}^{n}$ *is characterized as follows:*

(a) *It is monotone on* ${\mathbb{R}}^{n}$*, if for all* $𝔴,v\in {\mathbb{R}}^{n}$,

⟨Ψ(𝔴)−Ψ(v),𝔴−v⟩≥0.

(b) *It is strongly monotone on* ${\mathbb{R}}^{n}$ *with modulus* λ>0*, if for all* $𝔴,v\in {\mathbb{R}}^{n}$,

$$\langle \Psi (𝔴)-\Psi (v),𝔴-v\rangle \geq \lambda \|𝔴-v{\|}^{2}.$$

*Observe that the implication* (b)⟹(a) *is valid, but the reverse is not necessarily true in general. This indicates that monotonicity is a weaker property compared to strong monotonicity.*

**Definition 2.** [31] *The operator* $\Psi :{\mathbb{R}}^{n}\to {\mathbb{R}}^{n}$ *is called ζ-Lipschitz continuous on* ${\mathbb{R}}^{n}$ *if there exists a* ζ≥0*, such that*


$$\|\Psi (𝔴)-\Psi (v)\|\leq \zeta \|𝔴-v\|\;\forall 𝔴,v\in {\mathbb{R}}^{n}.$$

**Lemma 1.** [32] *Suppose that* 𝔇 *is a subset of* ${\mathbb{R}}^{n}$ *with non-empty, closed, and convex. Then, we obtain the following results: (a) In* (5)*, we defined* $P_{𝔇}$ *be the projection operator. Then*


$$\langle m-P_{𝔇}(m),P_{𝔇}(m)-n\rangle \geq 0,\;\forall m\in {\mathbb{R}}^{n},\;n\in {\mathbb{R}}^{n}.$$ (11)

*(b)* $P_{𝔇}(\cdot )$ *is a nonexpansive operator, if the following inequality holds:*


$$\|P_{𝔇}(m)-P_{𝔇}(n)\|\leq \|m-n\|,\;\forall m,n\in {\mathbb{R}}^{n}.$$ (12)

***Lemma 2.*** *For any* $a\in {\mathbb{R}}^{n}$, $b\in {\mathbb{R}}^{n}$*, the following inequalities holds:*

1. ⟨a,b⟩≤‖a‖‖b‖.
2. *For some* ϵ>0*, we have* $\|a\|\|b\|\leq \frac{\|a{\|}^{2}}{2\epsilon }+\frac{\epsilon \|b{\|}^{2}}{2}$.

**Theorem 1.** [14] *theorem 1 Suppose that* $\Phi :{\mathbb{R}}^{n}\to 2^{{\mathbb{R}}^{n}}$ *is a set-valued mapping whose values are nonempty, closed, and convex, and let* $\Psi :{\mathbb{R}}^{n}\to {\mathbb{R}}^{n}$ *be a* *λ*-*strongly monotone and* *ζ*-*Lipschitz continuous. In addition, suppose that there is a constant* ϱ>0 *such that*


$$\|P_{\Phi (m)}(z)-P_{\Phi (n)}(z)\|\leq \varrho \|m-n\|\;\forall \;m,n,z\in {\mathbb{R}}^{n},$$ (13)

*and the condition*


$$\sqrt{\zeta^{2}-2\mu \lambda +\mu^{2}}+\varrho <\mu ,$$ (14)

*holds for some constant* μ>0*, then IQVIP* (7) *admits a unique solution.*

**Definition 3** (Strong Global Solution). *We define $𝔴:{\mathbb{R}}^{+}\cup \{0\}\to {\mathbb{R}}^{n}$ as a strong global solution of the dynamical system* (9)*, if it satisfies the system’s equations for all s≥0 and meets the necessary conditions for existence and uniqueness over the set ${\mathbb{R}}^{+}\cup \{0\}$, and the following properties hold:*

1. *If we define the functions* 𝔴 *and* $\dot{𝔴}:{\mathbb{R}}^{+}\cup \{0\}\to {\mathbb{R}}^{n}$, *which means they are absolutely continuous on every interval* [0,q], *where* q>0.
2. $\ddot{𝔴}(s)+\tau (s)\dot{𝔴}(s)+\sigma (s)[\Psi (𝔴)-P_{\Phi (𝔴)}(\Psi (𝔴)-\mu 𝔴)]=0$ *for almost every* $s\in {\mathbb{R}}^{+}\cup \{0\}$.
3. $𝔴(0)={𝔴}_{0}$ and $\dot{𝔴}(0)=v_{0}$.

## 3 Main results

The subsequent result illustrates the existence and uniqueness of the trajectory for equation (9), with the proof adapted from [23]. Suppose $\Phi :{\mathbb{R}}^{n}\to 2^{{\mathbb{R}}^{n}}$ be a set-valued mapping such that each value is nonempty, closed, and convex throughout this paper.

**Theorem 2.** *If* $\sigma ,\tau :{\mathbb{R}}^{+}\cup \{0\}\to {\mathbb{R}}^{+}\cup \{0\}$ *are Lebesgue measurable functions, then* $\sigma ,\tau \in L_{\text{loc}}^{1}({\mathbb{R}}^{+}\cup \{0\})$ *(i.e.,* $\sigma ,\tau \in L_{\text{loc}}^{1}([0,c]$ *for each* 0<c<+∞)*. Consider the ζ-Lipschitz continuous operator of Ψ. Then, the dynamical system* (9) *has a unique strong global solution for each* ${𝔴}_{0},v_{0}\in {\mathbb{R}}^{n}$.

*Proof.* We define $A:{\mathbb{R}}^{n}\to {\mathbb{R}}^{n}$ by


$$A(𝔴)=\Psi (𝔴)-P_{\Phi (𝔴)}(\Psi (𝔴)-\mu 𝔴),\;\forall 𝔴\in {\mathbb{R}}^{n}.$$

Consequently, the dynamical system (9) can be alternatively represented as:


$$\left\{ \begin{array}{c} \ddot{𝔴}(s)+\tau (s)\dot{𝔴}(s)+\sigma (s)A𝔴(s)=0,\\ 𝔴(0)={𝔴}_{0},\;\dot{𝔴}(0)=v_{0}. \end{array}\right.$$ (15)

By the Cauchy-Schwarz inequality and Lemma 1(b), due to the Lipschitz continuity of Ψ, it follows that $\forall 𝔴,\bar{𝔴}\in {\mathbb{R}}^{n}$ and μ>0:


$$\begin{array}{c} \begin{array}{cc} \left\|A𝔴-A\bar{𝔴}\right\| & \;=\|\Psi (𝔴)-P_{\Phi (𝔴)}(\Psi (𝔴)-\mu 𝔴))-\Psi \bar{𝔴}+P_{\Phi (\bar{𝔴)}}(\Psi \bar{𝔴}-\mu \bar{𝔴}))\|\\ & \;\leq \|\Psi (𝔴)-\Psi \bar{𝔴}\|+\|P_{\Phi (𝔴)}(\Psi (𝔴)-\mu 𝔴)-P_{\Phi (\bar{𝔴)}}(\Psi (\bar{𝔴})-\mu \bar{𝔴}))\|\\ & \;\leq \|\Psi (𝔴)-\Psi \bar{𝔴}\|+\|P_{\Phi (\bar{𝔴})}(\Psi (𝔴)-\mu 𝔴)-P_{\Phi (𝔴)}(\Psi (\bar{𝔴})-\mu \bar{𝔴}))\|\\ & \;+\|P_{\Phi (𝔴)}(\Psi (\bar{𝔴})-\mu \bar{𝔴}))-P_{\Phi (\bar{𝔴)}}(\Psi (\bar{𝔴})-\mu \bar{𝔴}))\|\\ & \;\leq \zeta \|𝔴-\bar{𝔴}\|+\|\Psi (𝔴)-\mu 𝔴-\Psi (\bar{𝔴})+\mu \bar{𝔴})\|+\varrho \|𝔴-\bar{𝔴}\|\\ & \;\leq (\zeta +\varrho )\|𝔴-\bar{𝔴}\|+\|\Psi (𝔚)-\Psi (\bar{𝔴})\|+\mu \|𝔴-\bar{𝔴}\|\\ & \;\leq (\zeta +\mu +\varrho )\|𝔴-\bar{𝔴}\|+\zeta \|𝔴-\bar{𝔴}\|\\ & \;\leq (2\zeta +\mu +\varrho )\|𝔴-\bar{𝔴}\|. \end{array} \end{array}$$

Thus, *A* is Lipschitz continuous with modulus γ=2ζ+μ>0. Our dynamical system (15) can be alternatively reformulated as a first-order dynamical system within the product space ${\mathbb{R}}^{n}\times {\mathbb{R}}^{n}$:


$$\left\{ \begin{array}{c} \dot{X}(s)=𝔗(s,X(s)),\\ X(0)=(a_{0},v_{0}), \end{array}\right.$$ (16)

where $X:{\mathbb{R}}^{+}\cup \{0\}\to {\mathbb{R}}^{n}\times {\mathbb{R}}^{n}$, $X(s)=(𝔴(s),\dot{𝔴}(s))$, and


$$𝔗:{\mathbb{R}}^{+}\cup \{0\}\times {\mathbb{R}}^{n}\times {\mathbb{R}}^{n}\to {\mathbb{R}}^{n}\times {\mathbb{R}}^{n},\;𝔗(s,a,b)=(b,-\tau (s)b-\sigma (s)Aa).$$

We give ${\mathbb{R}}^{n}\times {\mathbb{R}}^{n}$ the scalar product $\left\langle (a,b),(\bar{a},\bar{b})\rangle_{{\mathbb{R}}^{n}\times {\mathbb{R}}^{n}}=\langle a,\bar{a}\rangle +\langle b,\bar{b}\right\rangle$ and the associated norm $\|(a,b{|}_{{\mathbb{R}}^{n}\times {\mathbb{R}}^{n}}=\sqrt{\|a{\|}^{2}+\|b{\|}^{2}}$.

By utilizing the Lipschitz continuity of *A*, we establish that for arbitrary $a,b,\bar{a},\bar{b}\in {\mathbb{R}}^{n}$ and ∀s≥0, i.e.,


$$\|𝔗(s,a,b)-𝔗(s,\bar{a},\bar{b}){\|}_{{\mathbb{R}}^{n}\times {\mathbb{R}}^{n}}=\sqrt{\|b-\bar{b}{\|}^{2}+\|\tau (s)(\bar{b}-b)+\sigma (s)(A\bar{a}-Aa){\|}^{2}}$$

$$\leq \sqrt{(1+2\tau^{2}(s))\|b-\bar{b}{\|}^{2}+2\gamma^{2}\tau^{2}(s)\|\bar{a}-a{\|}^{2}}$$

$$\leq \sqrt{\left(1+2\tau^{2}(s))+2\gamma^{2}\sigma^{2}(s\right)}\|(a,\bar{a})-(b,\bar{b}){\|}_{{\mathbb{R}}^{n}\times {\mathbb{R}}^{n}}$$

$$\leq (1+\sqrt{2}\tau (s))+\gamma \sqrt{2}\sigma (s)\|(a,\bar{a})-(b,\bar{b}){\|}_{{\mathbb{R}}^{n}\times {\mathbb{R}}^{n}}.$$

According to $\sigma ,\tau \in L_{\text{loc}}^{1}({\mathbb{R}}^{+}\cup \{0\})$, 𝔗(s,·,·) has a locally integrable Lipschitz constant. Now, we prove that


$$𝔗(\cdot ,a,b)\in L^{1}([0,c],{\mathbb{R}}^{n}\times {\mathbb{R}}^{n}),\;\forall a,b\in {\mathbb{R}}^{n},\forall c>0.$$ (17)

In fact, if c>0 and $a,b\in {\mathbb{R}}^{n}$ are arbitrary, then


$$\begin{array}{c} \begin{array}{cc} \int_{0}^{c}\|𝔗(s,a,b){\|}_{{\mathbb{R}}^{n}\times {\mathbb{R}}^{n}}dt & \;=\int_{0}^{c}\sqrt{\|b{\|}^{2}+\|\tau (s)b+\sigma (s)Aa{\|}^{2}}dt\\ & \;\leq \int_{0}^{c}\sqrt{(1+2\tau^{2}(s))\|b{\|}^{2}+2\sigma^{2}(s)\|Aa{\|}^{2}}dt\\ & \;\leq \int_{0}^{c}((1+\sqrt{2}\tau (s))\|b\|+\sqrt{2}\sigma (s)\|Aa\|)dt. \end{array} \end{array}$$

This integral is finite from the assumptions on σ,τ. By using the theorem of Cauchy-Lipschitz and Picard, we ensure the unique strong global solution for the system (16) in first-order (refer to Proposition 6.2.1 in [33] for more details). This conclusion arises from the equivalence between (9), (15), and (16).

### 3.1 Linear Convergence of a Discrete System

By applying a finite-difference scheme to equation (9) with respect to the time variable *s*, and using a step size $h_{k}>0$, a relaxation variable $\sigma_{k}>0$, a damping variable $\tau_{k}>0$, along with initial points ${𝔴}_{0}$ and $u_{1}$. Consequently, the iterative process can be expressed as follows:


$$\frac{1}{h_{k}^{2}}({𝔴}_{k+1}-2{𝔴}_{k}+{𝔴}_{k-1})+\tau_{k}\frac{1}{h_{k}}({𝔴}_{k}-{𝔴}_{k-1})=\sigma_{k}[P_{\Phi (𝔴)}(\Psi ({𝔴}_{k})-\mu {𝔴}_{k})-\Psi ({𝔴}_{k})].$$ (18)

We observe that, due to the Lipschitz continuity of $A𝔴:=\Psi (𝔴)-P_{\Phi (𝔴)}(\Psi (𝔴)-\mu \Psi (𝔴))$, there is some flexibility in its selection. Thus, we can express (18) as


$${𝔴}_{k+1}={𝔴}_{k}+(1-\tau_{k}h_{k})({𝔴}_{k}-{𝔴}_{k-1})+h_{k}^{2}\sigma_{k}[P_{\Phi (𝔴)}(\Psi ({𝔴}_{k})-\mu {𝔴}_{k})-\Psi ({𝔴}_{k})].$$ (19)

Setting $\theta_{k}=(1-\tau_{k}h_{k})$, $\rho_{k}=h_{k}^{2}\sigma_{k}$, the scheme can be reformulated as:


$${𝔴}_{k+1}={𝔴}_{k}+\theta_{k}({𝔴}_{k}-{𝔴}_{k-1})+\rho_{k}[P_{\Phi (𝔴)}(\Psi ({𝔴}_{k})-\mu {𝔴}_{k})-\Psi ({𝔴}_{k})].$$ (20)

This algorithm is a relaxed inertial projection and the convergence properties of (20) are examined in this section. Using the dynamical system (9), we examine the convergence of the trajectories 𝔴(s). The next result is essential for our convergence analysis.

**Theorem 3.** *Let* Ψ be *λ* *strong monotone and* *ζ*-*Lipschitz continuous on* ${\mathbb{R}}^{n}$. *Suppose that* ${𝔴}^{*}$ *be the unique solution of IQVIP* (7)*. If we denote* $z:=P_{\Phi (𝔴)}(\Psi (𝔴)-\mu 𝔴)$, $\forall \mu >0\text{ and }𝔴\in {\mathbb{R}}^{n}$,*. Then*


$$\langle 𝔴-{𝔴}^{*},z-\Psi (𝔴)\rangle \leq -\eta \|𝔴-{𝔴}^{*}{\|}^{2},$$ (21)

*and*


$$\begin{array}{c} \begin{array}{cc} \|\Psi (𝔴)-z{\|}^{2} & \;\leq -\frac{(2\zeta +\mu +\varrho {)}^{2}}{\eta }\langle 𝔴-{𝔴}^{*},z-\Psi (𝔴)\rangle . \end{array} \end{array}$$ (22)

*Proof.* From Lemma 1(a), we have the following,


$$\langle m-P_{\Phi (𝔴)}(m),P_{\Phi (𝔴)}(m)-n\rangle \geq 0,\;m\in {\mathbb{R}}^{n},\;n\in \Phi ({𝔴}^{*}).$$

Let m=Ψ(𝔴)−μ𝔴 and $z:=P_{\Phi (𝔴)}(\Psi (𝔴)-\mu 𝔴),$ we obtain


$$\langle n-z,\Psi (𝔴)-\mu 𝔴-z\rangle \leq 0,\;\forall n\in \Phi ({𝔴}^{*}).$$

Taking $n=\Psi ({𝔴}^{*})\in \Phi ({𝔴}^{*})$, we deduce that


$$\langle z-\Psi ({𝔴}^{*}),\Psi (𝔴)-\mu 𝔴-z\rangle \geq 0.$$ (23)

On the other hand, since ${𝔴}^{*}$ solves IQVI and the definition of $P_{\Phi (𝔴)}(\cdot )$ makes it evident that $z\in \Phi ({𝔴}^{*})$, we obtain


$$\langle z-\Psi ({𝔴}^{*}),\mu {𝔴}^{*}\rangle \geq 0.$$ (24)

Adding (23) and (24), we get


$$\langle z-\Psi ({𝔴}^{*}),\Psi (𝔴)-\mu (𝔴-{𝔴}^{*})-z\rangle \geq 0,$$ (25)

or equivalently,


$$\langle \mu (𝔴-{𝔴}^{*}),z-\Psi (𝔴)\rangle \leq -\langle \mu (𝔴-{𝔴}^{*}),\Psi (𝔴)-\Psi ({𝔴}^{*})\rangle -\|z-\Psi (𝔴){\|}^{2}+\langle \Psi (𝔴)-z,\Psi (𝔴)-\Psi ({𝔴}^{*})\rangle .$$ (26)

Using strong monotonicity of Ψ and the Cauchy-Schwarz inequality as well as Young’s inequality (for ϵ=1) in (26), we obtain


$$\langle \mu (𝔴-{𝔴}^{*}),z-\Psi (𝔴)\rangle \leq -\mu \lambda \|𝔴-{𝔴}^{*}{\|}^{2}+\frac{1}{2}\|\Psi (𝔴)-\Psi ({𝔴}^{*}){\|}^{2}.$$ (27)

Now, we use the Lipschitz continuity of Ψ in (27)


$$\langle \mu (𝔴-{𝔴}^{*}),z-\Psi (𝔴)\rangle \leq -\left(\mu \lambda -\frac{1}{2}\zeta^{2}\right)\|𝔴-{𝔴}^{*}{\|}^{2}.$$

Thus, we obtain


$$\langle 𝔴-{𝔴}^{*},z-\Psi (𝔴)\rangle \leq -\left(\lambda -\frac{1}{2\mu }\zeta^{2}\right)\|𝔴-{𝔴}^{*}{\|}^{2}.$$

or equivalently


$$\langle 𝔴-{𝔴}^{*},z-\Psi (𝔴)\rangle \leq -\eta \|𝔴-{𝔴}^{*}{\|}^{2}.$$

Also, based on conditions (13) and (14), Theorem 1 guarantees the existence and uniqueness of a solution to the IQVIP (7), denoted by ${𝔴}^{*}$. Furthermore, by utilizing the Lipschitz continuity of the function Ψ and the nonexpansiveness property of the projection operator, we obtain the following inequality for all $w\in {\mathbb{R}}^{n}$:


$$\begin{array}{c} \begin{array}{cc} \left\|(\Psi (𝔴)-z)-(\Psi ({𝔴}^{*})-z^{*})\right\| & \;=\|(\Psi (𝔴)-P_{\Phi (𝔴)}(\Psi (𝔴)-\mu 𝔴))-(\Psi ({𝔴}^{*})-P_{\Phi ({𝔴}^{*})}(\Psi ({𝔴}^{*})-\mu {𝔴}^{*}))\|\\ & \;\leq \|\Psi (𝔴)-\Psi \bar{𝔴}\|+\|P_{\Phi (𝔴)}(\Psi (𝔴)-\mu 𝔴)-P_{\Phi (𝔴)}(\Psi (\bar{𝔴})-\mu \bar{𝔴}))\|\\ & \;+\|P_{\Phi (𝔴)}(\Psi (\bar{𝔴})-\mu \bar{𝔴}))-P_{\Phi (\bar{𝔴)}}(\Psi (\bar{𝔴})-\mu \bar{𝔴}))\|\\ & \;\leq \zeta \|𝔴-\bar{𝔴}\|+\|\Psi (𝔴)-\mu 𝔴-\Psi (\bar{𝔴})+\mu \bar{𝔴})\|+\varrho \|𝔴-\bar{𝔴}\|\\ & \;\leq (\zeta +\varrho )\|𝔴-\bar{𝔴}\|+\|\Psi (𝔚)-\Psi (\bar{𝔴})\|+\mu \|𝔴-\bar{𝔴}\|\\ & \;\leq (\zeta +\mu +\varrho )\|𝔴-\bar{𝔴}\|+\zeta \|𝔴-\bar{𝔴}\|\\ & \;\leq (2\zeta +\mu +\varrho )\|𝔴-\bar{𝔴}\|. \end{array} \end{array}$$

Note that $\Psi ({𝔴}^{*})-P_{\Phi (𝔴)}(\Psi ({𝔴}^{*})-\mu {𝔴}^{*})=0$, combining (21) and above inequality, we obtain


$$\begin{array}{c} \begin{array}{cc} \|\Psi (𝔴)-z{\|}^{2} & \;\leq (2\zeta +\mu +\varrho {)}^{2}\|𝔴-{𝔴}^{*}{\|}^{2}\\ & \;\leq \frac{(2\zeta +\mu +\varrho {)}^{2}}{\eta }\langle \Psi (𝔴)-z,w-{𝔴}^{*}\rangle \\ & \;\leq -\frac{(2\zeta +\mu +\varrho {)}^{2}}{\eta }\langle w-{𝔴}^{*},z-\Psi (𝔴)\rangle . \end{array} \end{array}$$

**Theorem 4.** *Let Ψ be λ strong monotone and ζ-Lipschitz continuous on* ${\mathbb{R}}^{n}$. *Assume that*


$$\eta =\lambda -\frac{1}{2\mu }\zeta^{2}>0,$$

*and*


$$0<P<\rho_{k}C<Q,\;\text{where }C=(1+\theta_{k}^{2})$$ (28)

*or equivalently*


$$0<\frac{Q^{2}}{P}<\frac{2\rho_{k}C}{(2\zeta +\mu +\varrho {)}^{2}}.$$ (29)

*Then, the sequence* $\left\{{𝔴}_{k}\right\}$ *produced by the algorithm* (20) *converges linearly to the unique solution* ${𝔴}^{*}$ *of IQVIP* (7).

*Proof.* Suppose $\left\{{𝔴}_{k}\right\}$ be the sequence, which is generated by (20), we obtain


$$\begin{array}{c} \begin{array}{cc} \|{𝔴}_{k+1}-{𝔴}^{*}{\|}^{2} & \;=\|{𝔴}_{k}-{𝔴}^{*}+\theta_{k}({𝔴}_{k}-{𝔴}_{k-1})+\rho_{k}[P_{\Phi (𝔴)}(\Psi ({𝔴}_{k})-\mu {𝔴}_{k})-\Psi ({𝔴}_{k})]{\|}^{2}\\ & \;=\theta_{k}^{2}\|{𝔴}_{k}-{𝔴}_{k-1}{\|}^{2}+\|{𝔴}_{k}-{𝔴}^{*}+\rho_{k}[P_{\Phi (𝔴)}(\Psi ({𝔴}_{k})-\mu {𝔴}_{k})-\Psi ({𝔴}_{k})]{\|}^{2}\\ & \;+2\theta_{k}^{2}\langle ({𝔴}_{k}-{𝔴}_{k-1}),{𝔴}_{k}-{𝔴}^{*}+\rho_{k}[P_{\Phi (𝔴)}(\Psi ({𝔴}_{k})-\mu {𝔴}_{k})-\Psi ({𝔴}_{k})]\rangle . \end{array} \end{array}$$ (30)

We know that


$$\begin{array}{c} \begin{array}{cc} & \;\langle ({𝔴}_{k}-{𝔴}_{k-1}),{𝔴}_{k}-{𝔴}^{*}+\rho_{k}[P_{\Phi (𝔴)}(\Psi ({𝔴}_{k})-\mu {𝔴}_{k})-\Psi ({𝔴}_{k})]\rangle \\ & \;\leq \frac{1}{2}\|{𝔴}_{k}-{𝔴}_{k-1}{\|}^{2}+\frac{1}{2}\|{𝔴}_{k}-{𝔴}^{*}+\rho_{k}[P_{\Phi (𝔴)}(\Psi ({𝔴}_{k})-\mu {𝔴}_{k})-\Psi ({𝔴}_{k})]{\|}^{2}. \end{array} \end{array}$$ (31)

Therefore, from equations (30) and (31), we get


$$\begin{array}{c} \begin{array}{cc} \|{𝔴}_{k+1}-{𝔴}^{*}{\|}^{2} & \;\leq 2\theta_{k}^{2}\|{𝔴}_{k}-{𝔴}_{k-1}{\|}^{2}+(\theta_{k}^{2}+1)\|{𝔴}_{k}-{𝔴}^{*}+\rho_{k}[P_{\Phi (𝔴)}(\Psi ({𝔴}_{k})-\mu {𝔴}_{k})-\Psi ({𝔴}_{k})]{\|}^{2}\\ & \;\leq 2\theta_{k}^{2}\|{𝔴}_{k}-{𝔴}_{k-1}{\|}^{2}+(\theta_{k}^{2}+1)\|{𝔴}_{k}-{𝔴}^{*}{\|}^{2}\\ & \;+(\theta_{k}^{2}+1)\rho_{k}^{2}\|P_{\Phi (𝔴)}(\Psi ({𝔴}_{k})-\mu {𝔴}_{k})-\Psi ({𝔴}_{k}){\|}^{2}\\ & \;+2\rho_{k}(\theta_{k}^{2}+1)\langle {𝔴}_{k}-{𝔴}^{*},P_{\Phi (𝔴)}(\Psi ({𝔴}_{k})-\mu {𝔴}_{k})-\Psi ({𝔴}_{k})\rangle . \end{array} \end{array}$$ (32)

Setting $z_{k}=P_{\Phi (𝔴)}(\Psi ({𝔴}_{k})-\mu {𝔴}_{k})$ and combining with (22), it follows from (32) that


$$\begin{array}{c} \begin{array}{cc} \|{𝔴}_{k+1}-{𝔴}^{*}{\|}^{2} & \;\leq 2\theta_{k}^{2}\|({𝔴}_{k}-{𝔴}^{*})-({𝔴}_{k-1}-{𝔴}^{*}{\|}^{2}+(\theta_{k}^{2}+1)\|{𝔴}_{k}-{𝔴}^{*}{\|}^{2}\\ & \;-\frac{(2\zeta +\mu +\varrho {)}^{2}}{\eta }(\theta_{k}^{2}+1)\rho_{k}^{2}\langle {𝔴}_{k}-{𝔴}^{*},z_{k}-\Psi ({𝔴}_{k})\rangle \\ & \;+2\rho_{k}(\theta_{k}^{2}+1)\langle {𝔴}_{k}-{𝔴}^{*},z_{k}-\Psi ({𝔴}_{k})\rangle .\\ & \;=2\theta_{k}^{2}\|{𝔴}_{k}-{𝔴}^{*}{\|}^{2}-4\theta_{k}^{2}\langle {𝔴}_{k}-{𝔴}^{*},{𝔴}_{k-1}-{𝔴}^{*}\rangle +2\theta_{k}^{2}\|{𝔴}_{k-1}-{𝔴}^{*}{\|}^{2}\\ & \;+(\theta_{k}^{2}+1)\|{𝔴}_{k}-{𝔴}^{*}{\|}^{2}\\ & \;+(\theta_{k}^{2}+1)\rho_{k}\left(2-\frac{(2\zeta +\mu +\varrho {)}^{2}}{\eta }\rho_{k}\right)\langle {𝔴}_{k}-{𝔴}^{*},z_{k}-\Psi ({𝔴}_{k})\rangle . \end{array} \end{array}$$ (33)

Now, we applying $\langle {𝔴}_{k}-{𝔴}^{*},{𝔴}_{k-1}-{𝔴}^{*}\rangle \leq \frac{1}{2}\|{𝔴}_{k}-{𝔴}^{*}{\|}^{2}+\frac{1}{2}\|{𝔴}_{k-1}-{𝔴}^{*}{\|}^{2}$ in equation (33), we obtain


$$\begin{array}{c} \begin{array}{cc} \|{𝔴}_{k+1}-{𝔴}^{*}{\|}^{2} & \;\leq (\theta_{k}^{2}+1)\|{𝔴}_{k}-{𝔴}^{*}{\|}^{2}\\ & \;+(\theta_{k}^{2}+1)\rho_{k}\left(2-\frac{(2\zeta +\mu +\varrho {)}^{2}}{\eta }\rho_{k}\right)\langle {𝔴}_{k}-{𝔴}^{*},z_{k}-\Psi ({𝔴}_{k})\rangle . \end{array} \end{array}$$ (34)

We substitute the value from (21) in equation (34) to obtain


$$\begin{array}{c} \begin{array}{cc} \|{𝔴}_{k+1}-{𝔴}^{*}{\|}^{2} & \;\leq (\theta_{k}^{2}+1)\|{𝔴}_{k}-{𝔴}^{*}{\|}^{2}\\ & \;-\eta (\theta_{k}^{2}+1)\rho_{k}\left(2-\frac{(2\zeta +\mu +\varrho {)}^{2}}{\eta }\rho_{k}\right)\|{𝔴}_{k}-{𝔴}^{*}{\|}^{2}\\ & \;=(\theta_{k}^{2}+1)\left\{1-\eta \rho_{k}\left(2-\frac{(2\zeta +\mu +\varrho {)}^{2}}{\eta }\rho_{k}\right)\right\}\|{𝔴}_{k}-{𝔴}^{*}{\|}^{2}. \end{array} \end{array}$$ (35)

The last inequality follows from (28), (29) and (21). let $𝒢(\theta_{k},\rho_{k})$ be defined as


$$𝒢(\theta_{k},\rho_{k})=\sqrt{(\theta_{k}^{2}+1)\left\{1-\eta \rho_{k}\left(2-\frac{(2\zeta +\mu +\varrho {)}^{2}}{\eta }\rho_{k}\right)\right\}}\;\forall k>0.$$

Then (35) can be rewritten as


$$\|{𝔴}_{k+1}-{𝔴}^{*}\|\leq 𝒢(\theta_{k},\rho_{k})\|{𝔴}_{k}-{𝔴}^{*}\|.$$

After applying *n* iterations in the above inequality, we obtain the following result


$$\|{𝔴}_{k+1}-{𝔴}^{*}\|\leq \prod_{n=0}^{k}𝒢(\theta_{n},\rho_{n})\|{𝔴}_{0}-{𝔴}^{*}\|.$$ (36)

Let $C=(2-2\tau_{k}+\tau_{k}^{2})$. Then we have


$$𝒢(\theta_{k},\rho_{k}{)}^{2}=C-2\eta \rho_{k}C+(2\zeta +\mu +\varrho {)}^{2}C\rho_{k}^{2}.$$

Using the conditions (28) and (29), we get


$$𝒢(\theta_{k},\rho_{k}{)}^{2}<\frac{Q}{\rho k}-2\eta PC+(2\zeta +\mu +\varrho {)}^{2}CQ^{2}=r<1.$$ (37)

Combining (36) with (37), we obtain


$$0<\|{𝔴}_{k+1}-{𝔴}^{*}\|\leq r^{\frac{k}{2}}\|{𝔴}_{0}-{𝔴}^{*}\|.$$ (38)

As a result of equation (38), we can determine that the sequence $\left\{{𝔴}_{k}\right\}$ remains bounded. Additionally,


$$\|{𝔴}_{k+1}-{𝔴}^{*}\|\to 0,\text{ as }k\to \infty ,$$

which implies that $\left\{{𝔴}_{k}\right\}$ generated by the algorithm (20) converges strongly to solution ${𝔴}^{*}$.

**Corollary 1.** *Let Ψ be a monotone and ζ-Lipschitz continuous operator on* ${\mathbb{R}}^{n}$. *Assume that*


$$\eta =\lambda -\frac{1}{2\mu }\zeta^{2}>0,$$

*and*


$$0<P<\rho_{k}C<Q,\;\text{where }C=(1+\theta_{k}^{2}),$$ (39)

*or equivalently*


$$0<\frac{Q^{2}}{P}<\frac{2\rho_{k}C}{(2\zeta +\mu +\varrho {)}^{2}}.$$ (40)

*Then, the sequence* $\left\{{𝔴}_{k}\right\}$ *produced by the algorithm* (20) *converges linearly to the unique solution* ${𝔴}^{*}$ *of the IQVIP* (7).

## 4 Application of Lyapunov’s direct method for stability verification

Suppose $𝔛:{\mathbb{R}}^{n}\to \mathbb{R}$ be a Lyapunov function for the dynamical system (9), if it approximately characterizes the equilibrium point $𝔴={𝔴}^{*}$ and satisfies the following conditions:

(C1) The function 𝔛 is positive definite; that is, 𝔛(𝔴)≥0 for all $𝔴\in {\mathbb{R}}^{n}$, and 𝔛(𝔴)=0 ⇔ $𝔴={𝔴}^{*}$.

(C2) Along the trajectories of the dynamical system (9), the time derivative $\dot{𝔛}$ is negative definite. In other words, for any solution 𝔴(s) of (9), it holds that $\dot{𝔛}(𝔴(s))\leq 0$ for all s≥0, and $\dot{𝔛}(𝔴(s))<0$ whenever $𝔴(s)\neq {𝔴}^{*}$.

The following theorem represents a fundamental result in neurodynamic systems theory.

**Theorem 5.** *[Theorem of Lyapunov’s] Let ${𝔴}^{*}$ denote an equilibrium point of the dynamical system* (9)*. If the Lyapunov function is associated with ${𝔴}^{*}$, then it follows that ${𝔵}^{*}$ serves as a globally asymptotically stable state of the system* (9)*.*

We apply Theorem (5) to establish the stability of the solution corresponding to the system (9). Then, using Lyapunov’s direct method, we verify that the proposed second-order neurodynamic system (9) demonstrates asymptotic stability. For this purpose, we consider a Lyapunov candidate function defined as:


$$𝔛(𝔴)=\frac{1}{2}\left({\dot{𝔴}}^{2}+{𝔴}^{2}\right),$$

where 𝔛(𝔴) is clearly positive definite, i.e., 𝔛(𝔴)≥0, and 𝔛(𝔴)=0 ⇔ w=0. Thus, the derivative of the Lyapunov function 𝔛(𝔴) is:


$$\dot{𝔛}(𝔴)=\dot{𝔴}\ddot{𝔴}+u\dot{𝔴}.$$

Now, from the equation (9), we have


$$\ddot{𝔴}=-\tau (s)\dot{𝔴}-\sigma (s)[\Psi (𝔴)-P_{\Phi (𝔴)}(\Psi (𝔴)-\mu 𝔴)].$$

Now, substitute $\ddot{𝔴}$ into $\dot{𝔛}(𝔴)$, we obtain


$$\dot{𝔛}(𝔴)=\dot{𝔴}\left(-\tau (s)\dot{𝔴}-\sigma (s)[\Psi (𝔴)-P_{\Phi (𝔴)}(\Psi (𝔴)-\mu 𝔴)]\right)+𝔴\dot{𝔴}.$$

Simplifying, we get:


$$\dot{𝔛}(𝔴)=-\tau (s){\dot{𝔴}}^{2}-\sigma (s)\dot{𝔴}\cdot [\Psi (𝔴)-P_{\Phi (𝔴)}(\Psi (𝔴)-\mu 𝔴)]+𝔴\dot{𝔴}.$$

The dominant terms $-\tau (s){\dot{𝔴}}^{2}$ contributes to damping when τ(s)>0. The term $-\sigma (s)\dot{𝔴}[\Psi (𝔴)-P_{\Phi (𝔴)}(\Psi (𝔴)-\mu 𝔴)]$ ensures stability for σ(s)>0. The last term $w\dot{𝔴}$ represents interaction between position and velocity, then $\dot{𝔛}(𝔴)\leq 0$, ensuring Lyapunov stability $\dot{𝔛}(𝔴)$ is negative definite for all w≠0 in the Lyapunov sense. This implies that the equilibrium point ${𝔴}^{*}=0$ for the dynamical system (9) is globally asymptotically stable.

### 4.1 Numerical Illustration

In this section, two numerical examples are presented to demonstrate the effectiveness of the proposed dynamical system (9) in solving IQVIP (7).

**Example 1.** *Suppose the operator* $\Psi :{\mathbb{R}}^{3}\to {\mathbb{R}}^{3}$ *is defined as:*


$$\Psi (𝔴)=\left(e^{-0.5\|𝔴{\|}^{2}}+p\right)A𝔴,$$

*with parameters* p=2.5>0 *and* $A=[\begin{array}{ccc} 1.2 & 0 & -0.8\\ 0 & 1.8 & 0\\ -0.8 & 0 & 2.2 \end{array}]$ *be a symmetric matrix. Let* $𝔇=B[0,1]\subset {\mathbb{R}}^{3}$ *be the closed unit ball centered at the origin. then we defined* Φ(𝔴)=𝔴/4+𝔇.

**Fig 1:**
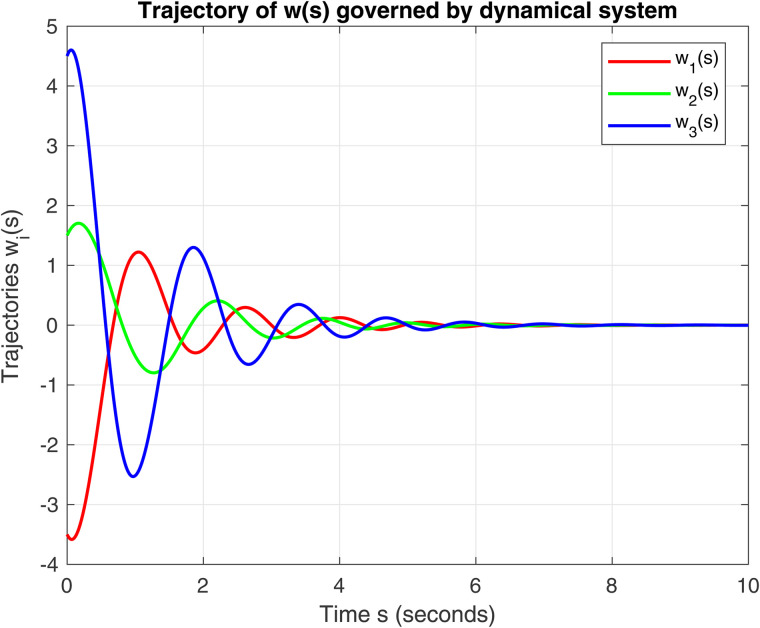
Evolution of trajectories for the initial conditions ${𝔴}_{0}=(-3.5,1.5,4.5{)}^{⊤}$ and $\dot{𝔴}(0)=v_{0}=(-2.5,2.5,3.5{)}^{⊤}$ in ${\mathbb{R}}^{3}$ using dynamical system (9).

**Fig 2:**
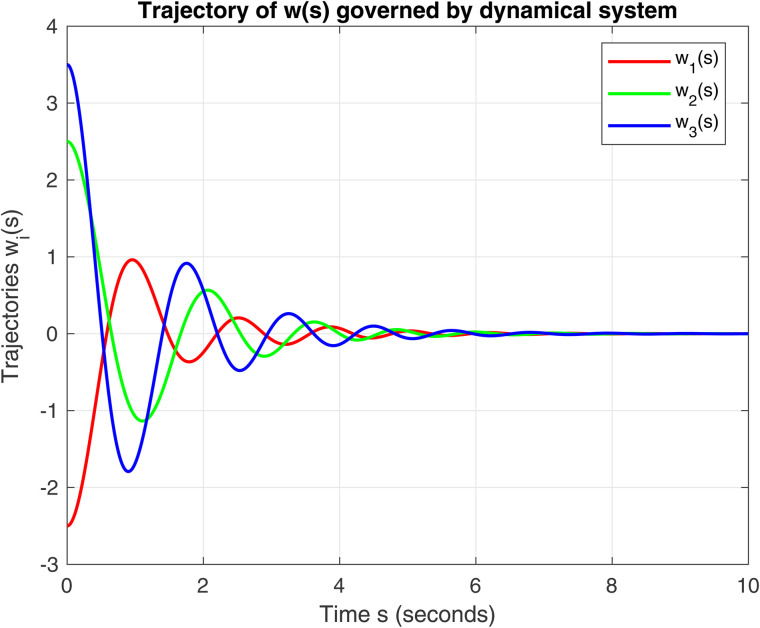
Evolution of trajectories for the initial conditions ${𝔴}_{0}=(-2.5,2.5,3.5{)}^{⊤}$ and $\dot{𝔴}(0)=v_{0}=(0,0,0{)}^{⊤}$ in ${\mathbb{R}}^{3}$ using dynamical system (9).

The unique solution to the variational inequality IQVI is ${𝔴}^{*}=(0,0,0{)}^{⊤}.$ The parameters for this dynamical system (9) are σ=1.8, τ=1.5, μ=0.85, ζ≈5.5, η=1 and $\mu_{min}=\text{min}(eig(A))$ (smallest eigenvalue of *A*), then $\lambda =p.\mu_{min}$ and $\mu =\frac{\zeta^{2}}{2(\lambda -\eta )}$. The numerical solution confirms that the trajectories of $w_{1}(s),w_{2}(s),w_{3}(s)$ globally converge to ${𝔴}^{*}$. The numerical behavior is illustrated in Figs 1 and 2. Fig 1. This is the first figure of Example 1. Fig 2. This is the second figure of Example 1.

**Example 2.** *Suppose the operator* $\Psi :{\mathbb{R}}^{6}\to {\mathbb{R}}^{6}$ *is defined as:*


Ψ(𝔴)=(cos(0.4‖𝔴‖)+p)A𝔴,

*with parameters* p=1.8>0 *and*


$$\begin{array}{c} A=[\begin{array}{cccccc} 2 & 0.3 & 0 & 0 & 0 & -0.2\\ 0.3 & 1.7 & 0 & 0.2 & 0 & 0\\ 0 & 0 & 2.1 & 0 & 0.1 & 0\\ 0 & 0.2 & 0 & 1.9 & 0 & 0.3\\ 0 & 0 & 0.1 & 0 & 1.8 & 0\\ -0.2 & 0 & 0 & 0.3 & 0 & 1.6 \end{array}] \end{array}$$

*be a symmetric matrix. Let* $𝔇=\{𝔴\in {\mathbb{R}}^{6}:{𝔴}^{⊤}Q𝔴\leq 1\},\;Q=diag(1,0.9,0.7,0.5,0.6,0.4),$ *then we define the set-valued map* $\Phi (𝔴)=\frac{𝔴}{5}+𝔇$. *The parameters are* ζ=5.5, η=1.0*, and compute* $\mu_{\min}=\min(eig(A))$*. Also, we assume that the time-dependent coefficients for the dynamical system* (9) *are* $\tau (s)=1.3+0.3e^{-0.05s},\;\sigma (s)=1.5+0.2\sin(0.2s)$*. Then choose*


$$\lambda =p\cdot \mu_{\min},\;\mu =\frac{\zeta^{2}}{2(\lambda -\eta )}.$$

**Fig 3:**
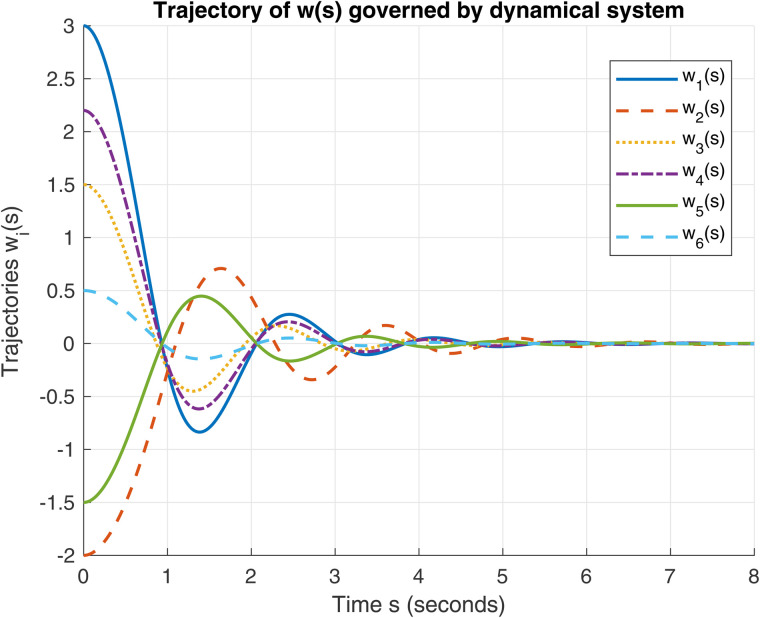
Evolution of trajectories for the initial conditions ${𝔴}_{0}=(3,-2,1.5,2.2,-1.5,0.5{)}^{⊤}$ and $\dot{𝔴}(0)=v_{0}=(0,0,0,0,0,0{)}^{⊤}$ in ${\mathbb{R}}^{6}$ using dynamical system (9).

**Fig 4:**
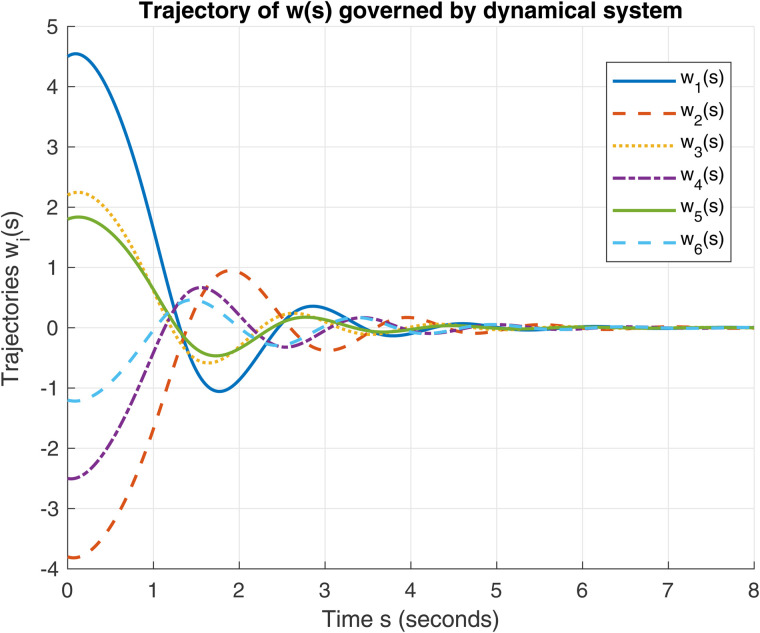
Evolution of trajectories for the initial conditions ${𝔴}_{0}=(4.5,-3.8,2.2,-2.5,1.8,-1.2{)}^{⊤}$ and $\dot{𝔴}(0)=v_{0}=(1.0,-0.5,0.8,-0.3,0.6,-0.4{)}^{⊤}$ in ${\mathbb{R}}^{6}$ using dynamical system (9).

*The unique solution of the corresponding IQVI is* ${𝔴}^{*}=0$*. Numerical simulations indicate that each component* $w_{i}(s)$ (i=1,…,6*) converges smoothly to zero as* s→∞*, confirming global convergence of the system. The numerical behavior is illustrated in* Figs 3 *and* 4. *Fig 3 This is the first figure of Example 2. Fig 4. This is the second figure of Example 2.*

## 5 Conclusion

This paper introduces a second-order dynamical system for solving IQVIP involving a strongly monotone and Lipschitz continuous operator. Under suitable parameter conditions, we establish the existence and uniqueness of strong global solutions, ensuring that the proposed continuous-time model is mathematically well-posed and robust.

Moreover, we proved that the discrete sequence generated by the relaxed inertial projection scheme corresponding to the dynamical system (9) converges linearly to the unique solution of the IQVIP (7). The corresponding discrete algorithm achieves linear convergence, supported by the theoretical conditions of our main theorem. The Lyapunov-based analysis establishes global asymptotic stability of the system (9), ensuring reliability and robustness of the approach. Finally, numerical illustration confirms that all trajectories of the proposed dynamical system converge smoothly to the unique solution of the IQVIP (7), demonstrating both the existence and stability of the solution. Overall, the proposed framework deepens the connection between IQVIP and dynamical system techniques, offering a foundation for further development in optimization and nonlinear analysis.

Future work includes extending this approach to higher-order systems and refining stability criteria for broader applications. These findings contribute to the analysis and computation of dynamical systems in Hilbert spaces.
